# Boston Letter

**Published:** 1879-02

**Authors:** 


### Domestic Correspondence.

### ARTICLE XIII.

### BOSTON LETTER.

MESSRS. EDITORS:—The profession of medicine in Boston has a strong but quiet flow. Much goes on which comes to the knowledge of but few outside of certain circles. It is almost odd that here there is a lack of that medical gossip which is a strong, a pleasant and a useful characteristic in other cities. I suppose there is no city in the country in which medicine is more reserved and retiring than in Boston. We are decidedly and essentially conservative. It might be a difficult matter to discover the reason of this—to trace back to its original causes the very calm tone of the medical mind in this city. It doubtless would be still more difficult to say why it is that in this fruitful age of medicine, the men here who carry the responsibilities which are laid upon them by great opportunity and wealth of material, are so silent toward that great world of medicine which awaits, expects and longs for revelations from the comparative few who hold the golden key to a treasure house which here is opened, not freely, not generously, but only to a favored few. In justice to the younger men, however, and notably the specialists, I should say that this surprising conservatism is almost wholly a fault, for it is a fault, of the older men. The young physicians of Boston have plenty of that sort of enthusiasm which leads one at once to impart to the profession at large, not only all new facts, new data, new effects of medicines, new discoveries in medical science, but also any modifications of facts already known. But they do this in spite of a chilling influence which arises from the marked reserve of the older men, many of whom rarely touch a pen in behalf of medical publications, so that much which would benefit our common cause remains unwritten, and but for the students and occasionally the medical societies of Boston, would likewise remain unspoken and unrevealed. Why is this ? Is it apathy? Is it lack of a sympathetic desire to advance the medical knowledge of the many ? Is it heedlessness of responsibility ? None of these reasons explain the facts; for the interest of these men in all that pertains to medicine is deep and intense. The kindest explanation of this reserve and silence is, that habit is the cause, habit which has become more powerful than the desire to break it. And yet the men to whom I refer are men of great ability, of unusual acumen, of large intelligence and profound learning. They should be known throughout the country, but with some exceptions they are not known to any degree out of the State. If this seem exaggeration, I would ask you, with how many standard, original medical works you are familiar which were written in Boston within the past twenty-five years ? It is weakness in a medical man to write a book unless he have something new to say, or a new and better method of presenting a known subject. But it is impossible that men who are mature in knowledge, rich in experience, hard students, keen diagnosticians, and who control and handle a vast amount of material, have not something to say which would increase the knowledge of every man who read or heard it. If one see a common fault which might easily be removed, should he not frankly bring it into notice, especially if he do so with the sincere and earnest hope that his frankness may be suggestive ? This is my apology for bluntness in alluding to men whose power and ability have won my sincerest respect, and whose light, let us hope, will be set upon the house-top before they depart. For, every mind of power should work not only for the present but also in behalf of posterity.

On the other hand, we point with pride to those reports on the progress of medicine, surgery, and kindred branches which are published in the *Boston Medical and Surgical Journal*, and which are accumulating in quantity and value. Published in book form they would be invaluable for reference. These reports are the work of young men, who are not mere harvesters of the fields of other men's planting, but whose intelligent and critical opinions and experience increase the usefulness of this history of the growth of medicine in all its relations. Probably, then, in the coming 25 years, this habit of writing on the part of young men will give a far different complexion to the future medical history of Boston from that which has characterized it during the past quarter of a century. After completing their studies at the Harvard Medical School, quite a large proportion of the city men go abroad. Indeed, where the means to do so are forthcoming, a year or two in the German schools is rather the rule than the exception. The effect of this has been apparent for some years in the more generous recognition of specialties here than is the case in other cities. But it can be said with perfect truth, that specialists in Boston are not confined to the narrow path of their choice, for in almost every instance there exists a good, solid foundation of experience which has been derived from hospital and general practicc.

This removes a large share of that prejudice against specialties which is a common feeling of the older medical men everywhere. And it must be confessed that without a foundation of general medical knowledge, the pursuit of a single branch of medical science, has a tendency not only to make a man narrow, but its influence is liable to give him a one-sided view of his cases.

I think I may say that our medical societies compare favorably with those of other American cities, for, if our older men do not write, many of them are faithful attendants of, and active participants in, our Society meetings. Their presence gives a firmer and maturer tone to discussions, and exerts a wholesome influence upon younger men of lax tendencies. Our most democratic meeting is that of the Suffolk District Society, a branch of the State Society. It is attended by every class of medical mind, while the smaller associations, the Society for Medical Improvement and the Society for Medical Observation, are composed of men who, for the most part, are constant students and steady workers; thinkers—men who have opinions and are able to express them. The first of these societies devotes itself mainly to pathology and possesses a fine anatomical collection, now in keeping of the Medical School. The other occupies itself with clinical discussion and research. In the Suffolk District Society, any subject which has a direct or indirect bearing upon medicine is in order. There are various other societies which I will not at present mention. But I may say that in the Harvard Medical School is an organization of great educational value. This is the Boylston Medical Society—composed of students, presided over by a physician who is elected by the active members, the object of the Society being to stimulate emulation and inquiry among the students. It was organized in 1811. The beneficial effects of this association are at once apparent. The members become habituated to discussion of medical topics; they become familiar with parliamentary laws; they learn how to prepare papers, and since the subject of papers which are read at the meetings are announced beforehand, the active and ambitious are led to read and make themselves ready for discussion. By this means, too, they are prepared to do good work in the societies of which they may become members in the future. This society should be imitated wherever medicine is taught. There are other features of medical teaching in the Harvard School, which not only make it the most desirable school in the country, but which are peculiar to it. In a future letter I will give you interesting details of the management of this department of Harvard University. Its adoption of a higher and more stringent standard of education was a bold move. It was an indication of the purity of the desire of the faculty of teachers to raise the educational level of the school, and to improve the quality of their graduates—for, in order to accomplish this desire, every man of the faculty was obliged to make personal sacrifice by yielding a portion of his salary. If this be not an evidence of sincerity, show me a better. Everybody knows that the great stumbling block in the upward path of other successful medical schools, is the fear lest the yearly income become smaller. This hyperæsthesia of the pocket nerve is what prolonged the discussion of the question of higher standards at the University of Pennsylvania, and which keeps other medical schools in the trammels of the old-fashioned, unsystematic olla podrida plan of teaching. So far as the Harvard School is concerned, the change has been a perfect success. The quality of the students has become vastly improved, and the value of the dioploma, of course, is above that of any other American school. For while the standard at the Medical School of the University of Pennsylvania has been raised. there is no preliminary examination, and other requirements are not so strict as in our school. In making the change in the plan of education, the authorities of the Harvard School were obliged to assume risks which Dr. Henry J. Bigelow tersely expressed as follows: 1. "The reduction of the salaries of the professors. 2. Reduction of the hole of escape for students. 3. Putting the students through a preliminary sieve." The possible effect of these conditions, however, was cordially and bravely assumed, with results which simply make us all proud of the school which never has been more successful. Moreover, it is so conducted, the opportunities for clinical and surgical teaching are so wisely and shrewdly used, and such a variety of specialties stands upon the roster, that the necessity for going abroad is no longer urgent. Students can not only become accomplished in any branch of medicine they may elect, but are not obliged to leave the school upon graduation, for they may at once enter upon the "post graduate course," which is open to older physicians as well as to recent graduates, and comprises *fourteen* special studies taught by accomplished men who have have received special training. It is a matter of surprise that the *New York Medical Record* was not aware of this course. In the edition of December 28, an editorial in that journal gravely suggests that the time has come for some school to take the "initiative" in giving lectures and instructions to physicians. This plan has been successfully pursued at the Harvard School for several years. The faculty is very much in earnest in giving the school the most modern methods of teaching, and in acquainting its students with every new and approved fact in medical science. These remarks are merely introductory to much which I shall have to say hereafter.

Within a few days we have followed to the grave two Boston physicians of high fame, Dr. Jacob Bigelow, father of Dr. Henry J. Bigelow, and once so active as teacher, author, physician, and scientist, and Dr. J. B. S. Jackson, the distinguished pathologist. Dr. Bigelow was formerly a Harvard professor; was ex-president of the American Medical Association; was long a president of the American Society of Arts and Sciences, and was the oldest member of the Massachusetts Medical Society. His work on "American Medical Botany;" his work on "Nature in Diseases," and other medical and literary publications are well known. It was to his influence that the inauguration of our excellent Institute of Technology was mainly due. Indeed he was the first to bring into use the term technology, in its present signification. He was also the wise founder of our beautiful Mount Auburn Cemetery, whose architectural beauties were designed by him and which has served as a model for similar places in our country. Dr. Bigelow has lived so long in retirement that many of the present generation never have seen him.

He died at the unusual age of ninety-one years, following close upon the steps of a man whose age was venerable but who was so much younger than Dr. Bigelow, that he was born in the same year in which Dr. Bigelow graduated from Harvard. Jackson died at the age of seventy-two. He was a pathologist of such enthusiasm that his whole life has been devoted to his beloved specialty. For many years he has been professor of pathology in the Harvard School. He was a pathologist of the old school. He was not a microscopist. But his unaided eye often told the truth when the microscope failed. His life was bound up in the Warren Anatomical Museum and the Cabinet of the Society for Medical Improvement. His catalogue of the former is an octavo volume of 750 pages. That of the latter amounts to 350 pages. The earnest faithfulness with which he devoted himself to the arrangement and description of these two collections cost him half a life time, but the museums have a world-wide fame, and they form a lasting monument to the profound pathological knowledge of Dr. Jackson.

BOSTON, Jan. 22, 1879.

